# Characterization of Modified Tapioca Starch Solutions and Their Sprays for High Temperature Coating Applications

**DOI:** 10.1155/2014/375206

**Published:** 2014-01-28

**Authors:** M. Y. Naz, S. A. Sulaiman, B. Ariwahjoedi, Ku Zilati Ku Shaari

**Affiliations:** ^1^Department of Fundamental and Applied Sciences, Universiti Teknologi PETRONAS, Bandar Seri Iskandar, 31750 Tronoh, Perak, Malaysia; ^2^Department of Mechanical Engineering, Universiti Teknologi PETRONAS, Bandar Seri Iskandar, 31750 Tronoh, Perak, Malaysia; ^3^Department of Chemical Engineering, Universiti Teknologi PETRONAS, Bandar Seri Iskandar, 31750 Tronoh, Perak, Malaysia

## Abstract

The objective of the research was to understand and improve the unusual physical and atomization properties of the complexes/adhesives derived from the tapioca starch by addition of borate and urea. The characterization of physical properties of the synthesized adhesives was carried out by determining the effect of temperature, shear rate, and mass concentration of thickener/stabilizer on the complex viscosity, density, and surface tension. In later stage, phenomenological analyses of spray jet breakup of heated complexes were performed in still air. Using a high speed digital camera, the jet breakup dynamics were visualized as a function of the system input parameters. The further analysis of the grabbed images confirmed the strong influence of the input processing parameters on full cone spray patternation. It was also predicted that the heated starch adhesive solutions generate a dispersed spray pattern by utilizing the partial evaporation of the spraying medium. Below 40°C of heating temperature, the radial spray cone width and angle did not vary significantly with increasing Reynolds and Weber numbers at early injection phases leading to increased macroscopic spray propagation. The discharge coefficient, mean flow rate, and mean flow velocity were significantly influenced by the load pressure but less affected by the temperature.

## 1. Introduction

Starch is a natural polymeric product and is found in almost every plant. Today, the main sources of most of the commercially available starches are tapioca, potato, maize, rice, wheat, and so forth. The chemical makeup of these starches makes them very good adhesives for many industrial processes including paper boxes, paper bags, corrugated box boards, laminated paper boards, case sealing, carton sealing, tube winding, gummed paper, gummed tape, and textile sizing. In recent years, the research interests in starches have widened from their global food ingredient to many industrial and technological applications including starch based biodegradable materials for the pharmaceutical industry and agricultural practices [1]. For example, up till now, the main goal of applying fertilizers was to provide nutrients to plants to increase or sustain optimal crop yield. Nitrogen, due to its mobility in the soil, loses by volatilization and leaching which result in marked inefficiency, less economic cultivation, reduced biomass production, and adverse environmental impacts. Therefore, the researchers and fertilizer producers have been trying to find ways to achieve the newly defined goal of fertilizer uses. In this connection, the technology of coating urea granules by liquid polymers is acquiring wide recognition by advanced growers of horticultural crops worldwide. Being cheap, biodegradable, and environmentally friendly biopolymers, the starch adhesives may also be used as a potential material to coat the urea granules for controlled release of nitrogen into the field [2].

For efficient granules coatings, the proper design of starch adhesives and associated coating mechanism is of great importance. The pure starches do not meet the standards set by many industrial processes due to slow tacking and too low viscosities. But many adhesives derived from pure starches can significantly be improved by urea derivatives and borate additives through viscosity enhancement, quick tacking, and better solution properties [3]. Extensive physical and chemical changes (cross-linking, interchain linkages) are possible by treating the starches with urea and sodium borate. These additives can change the pure starch into a highly branched long chain biopolymer of improved viscosity, tacking, and solution properties [4].

It is well known that not only the solution physical properties but also the associated coating mechanism is of great importance for successful coating processes. The air-assisted and airless/hydraulic sprays are most commonly used mechanics in coating industry. The starches modified with urea and borate additives are highly viscous and non-Newtonian in nature; therefore, the control over physical properties and then atomization into fine droplets spray would be a challenge for researchers. There is a considerable amount of information available in the literature relating to the phenomenological studies on the effects of the chemical additives and physical properties of the Newtonian solutions [5–7]. There is no systematic study on the effects of the chemicals additives, physical properties, and nozzle design on atomization patterns of biodegradable non-Newtonian solutions. Furthermore, the practical spray nozzles are designed for use in water systems and manufacturers characterize them using water. These nozzles are then used with complex and non-Newtonian solutions containing many different chemicals. The resultant combination of such solutions and spraying systems then often fails to meet the manufacturer's specification. More probably, it might be due to the fact that non-Newtonian solutions are being pushed through the spraying systems that are designed for Newtonian solutions. Therefore, further research work on gaining an insight into this aspect of practical spray coating systems would be of great use in future agricultural industry [8].

The present work with the specific objectives places greater emphasis on designing of the starch adhesives and phenomenological analyses of their spray jet breakup under varying pumping pressure and heating temperature in still environment, where the dimensionless spray parameters and aerodynamic effects are anticipated to be more important in resolving the limitations of the axisymmetric jet breakup mechanism. Therefore, in this detailed study, three starch adhesives of different mixing ratios were prepared for future coating practices. The effect of temperature, shear rate, and borate mass concentration on their physical properties was investigated. Finally, in view of these properties, the starch adhesives were heated and sprayed into full cone spray patterns using a laboratory scale intermittently forced liquid spraying mechanism. The solution heating was carried out for momentarily lowering the adhesive viscosity, surface tension, and load pressure and for atomizing it into fine droplets spray patterns. The corresponding spray patterns were visualized by a high speed camera at different frame rates. The generated data was used to determine important spray parameters like mass flow rate, nozzle discharge coefficient, Weber number, Reynolds number, spray cone angle, cone length, cone width, and jet penetration for different solution temperatures and pumping pressures.

## 2. Materials and Methods

### 2.1. Synthesis of Starch Adhesives

Three compositions of starch-urea-borate complex solution were prepared by following the procedure of Ariyanti et al. after some modifications [9]. The composition of the materials in g/1000 mL of water is stated in Table 1. First of all, the tapioca starch was dried at 110°C for 2 hours in order to remove all the moisture contents or until no further weight transformations were noticed. The measured quantity of fully dried tapioca starch was put in a stainless steel iron-mixed container and dissolved in water. For complete gelatinization, the solution was reacted at 80°C for 30 minutes. The solution heating was carried out using a DIPO induction cooker (TCK35-E) equipped with a temperature sensor. An overhead stirrer (IKA-RW 20) was also used for stirring and uniform mixing of solution at 600 rpm. After 30 minutes, the known masses of urea (cross-linker) and borate (gelling agent) were added into starch dispersion. The solution was then heated further for 3 hours at a constant temperature of 80°C and 600 ± 5 rpm stirring. The final solution was left to cool to room temperature before further characterization.

### 2.2. Measurement of Physical Properties

After 24 hours cooling, the quality of synthesized starch adhesives was ascertained by investigating their physical properties like dynamic viscosity, surface tension, and density. The dynamic viscosity was measured using OFITE-1100 viscometer and ORCADA software at a solution temperature range of 20–100°C and shear rate range of 1–1000 s^−1^. This all in one viscometer design includes the Couette geometry, heating mechanism, bobs, and rotor for viscosity measurements. For accurate measurements, the sample temperature was kept constant for at least 5 minutes before each run. The surface tension was measured using the pendant drop method as reported in Skurtys and Aguilera [10]. The density of all the prepared samples was measured as a function of heating temperature by using an Anton Paar DMA 4500M density meter.

### 2.3. Spraying System Specification

**Figure 1:**
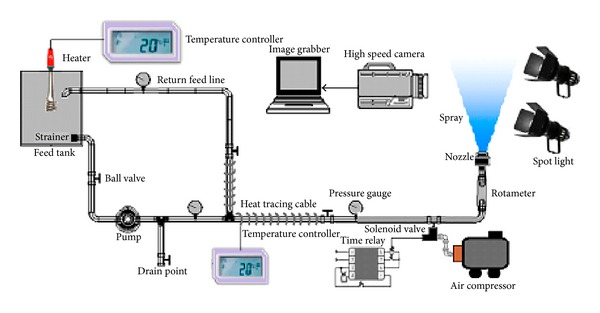
Schematic of the experimental spray rig.

After collecting the data on physical properties of all three compositions, the starch adhesives were sent to the spraying system where they were atomized into full cone spray patterns. The experimental setup used for spray generation and visualization is shown in Figure 1. The spraying mechanism was to produce bottom to top full cone spray patterns in a pulsed manner. For this purpose, an axisymmetric full cone spray nozzle from RELAB having an orifice diameter 1.19 mm and maximum free passage diameter 0.64 mm was used to spray the starch adhesive solutions at elevated temperature and pressure. This axisymmetric nozzle was equipped with a special X-shaped vane fixed at the nozzle inlet to impart the swirl and rotational speed to produce a full cone spray pattern. The spray pulse on-off duty cycle was controlled by a system composed of a PROVAL pneumatic double actuated solenoid valve and a programmable digital time relay (SIGMA, PTC-15). This solenoid valve releases the pressurized water in pulsed mode from spraying nozzle only when activated and avoids the overspray. The valve duty cycle was kept constant at 1.2 seconds throughout the course of current experiment. In order to maintain a desired high temperature within the feed tank and spray feed line to spray point, the liquid immersion heater and heating tracing cables were used and the corresponding water temperature was monitored using thermostatic controllers. The service temperature was elevated from 20 to 100°C for momentarily lowering the required load pressure, viscosity, and surface tension and to ease the liquid delivery process. In order to avoid the thermal losses, the heat tracing cable was insulated using the ceramic tape. In addition to temperature, the service pressure was being elevated from 0.5 to 1.5 bar. For this purpose a liquid delivery pump capable of withstanding high temperature values was used to serve the spray nozzle at its required spraying pressure. In order to avoid the overload and pump damaging situations, a return feed line was connected to the main system to drain out excess liquid back into the feed tank for recirculation. A stainless steel mesh strainer hampering any possible tiny debris from the liquid flow was also fixed at the feed tank outlet. The liquid pressure at three different localized points in main supply line was monitored using spring type pressure gauges and a drain pipe was introduced for the ease of flushing the leftover solution within the supply line after each operation.

### 2.4. Spray Visualization and Measurements

In order to characterize the sprays of the tested solutions, the input parameters like temperature, pressure, liquid flow rate, pulse duration, and dynamic viscosity were varied; the corresponding spray characteristics including the axial spray tip penetration, jet breakup mechanism, spray cone angle, spray width, nozzle discharge coefficient, Weber number, and Reynolds number were determined using flow measuring and nonintrusive image visualization tools. The visualization system used in this experiment was composed of a high speed digital camera and a spray chamber side illuminating arrangements. The transparent spray chamber was illuminated from all sides using 9 spotlights of 300 Watts each and the spray jet movements were visualized using a Phantom v9.1 digital camera. This 14 bit and 2 megapixels high speed camera suites well to the larger field of view applications. It was capable of recording the 1,016 fps with a resolution of 1632 × 1200 pixels and simultaneously transferring the captured cine files to an image grabber. At a time, it can hold up to 24 GB of images. The high definition camera output also provided the immediate playback of the recorded movies and online picture display [5–7].

Using this camera, the different spray parameters have been investigated for each flow condition using frame by frame image processing method followed by manual verifications. The camera generated data has been analyzed using MATLAB and other visual softwares for the study of some of the most important spray parameters including the axial spray tip penetration, jet breakup mechanism, spray cone angle, spray width, nozzle discharge coefficient, Weber number, and Reynolds number. In these investigations, rather using a commercially available flowmeter, a manual method was used to measure the water flow rate while the mean flow velocity was determined by dividing the water flow rate by cross-sectional area of the nozzle orifice. The discharge coefficient, Weber number, and Reynolds number were determined by using the equations

(2.4) CD=v2Δp/ρ,We=ρdv2σ,Re=ρdvμ,

where *v* is the mean flow velocity at nozzle exit, Δ*p* is the pressure difference, *d* is the nozzle exit diameter, *ρ* is the water density, *σ* is the surface tension, and *μ* is the dynamic viscosity.

## 3. Results and Discussion

### 3.1. Adhesive Physical Properties 

#### 3.1.1. Gelatinization Temperature

The temperature at which pure starch granules in heated water dispersion undergo transitions from the crystalline to a gel state is called gelatinization temperature. It is considered as an important parameter while characterizing the starches and their solutions under certain defined conditions. It helps in determining the starch type and heterogeneity in granule population. The individual starch granules have a range of gelatinization temperatures and undergo gelatinization at a specific temperature within the range. In these investigations, the 5% aqueous dispersion of tapioca starch was gelatinized in the low temperature range of 63–84°C. It has been reported in the literature that the gelatinization temperature range depends on difference in degree of crystallites heterogeneity within the granules [11]. Therefore, the gelatinization range of tapioca starch reveals higher heterogeneity in crystallites of its granules and consequently the varying tightness of granule structure [12].

#### 3.1.2. Rheological Properties

**Figure 2:**
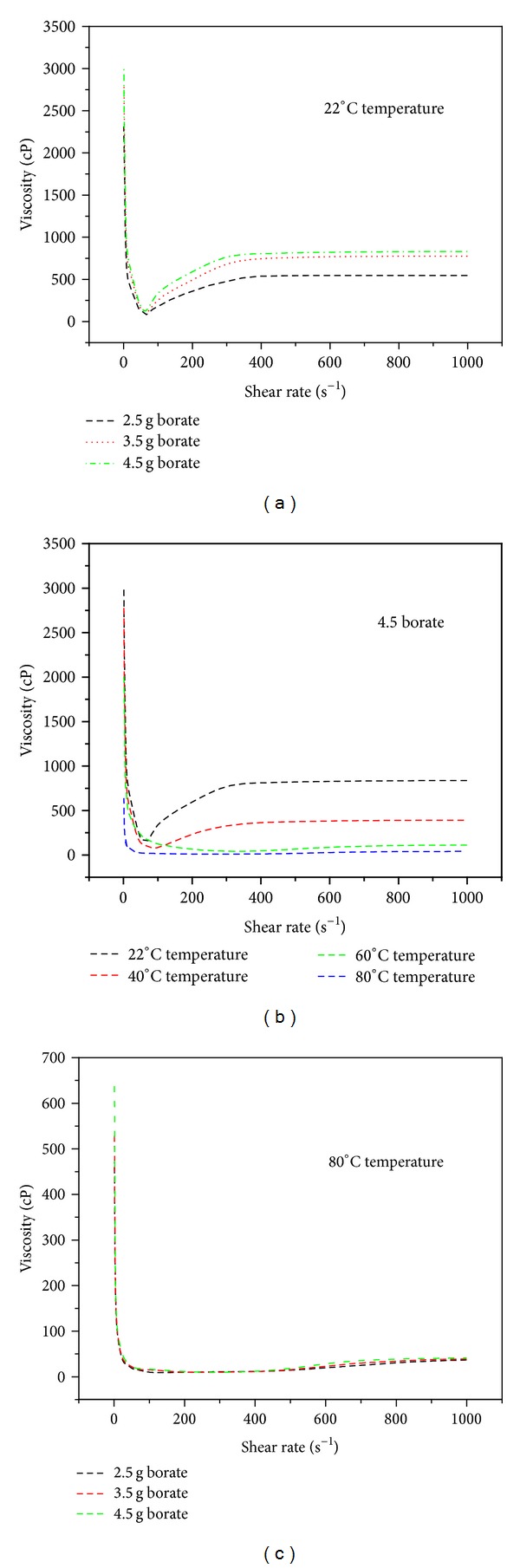
(a) Starch adhesive viscosity as a function of applied shear rate for different borate concentrations. (b) Starch adhesive viscosity as a function of applied shear rate for different temperatures. (c) Starch adhesive viscosity as a function of the applied shear rate at 80°C fixed temperature.

In coating applications, the viscosity of the starch adhesives is very important for quality coatings. The chemical thickeners that are used for production of starch adhesive can either be an acidic or basic in nature. In these studies, the viscosity effects of borate as a viscosity thickener were investigated. The adhesives produced at 80°C were found to have very promising results when compared with the commercially available adhesives [1–4]. Hence, this temperature was used to produce three starch adhesives having 2.5, 3.5, and 4.5 g mass concentration of the borate modifier. The effect of the modifier concentration on the adhesive viscosity, density, and surface tension was investigated. The viscosity as a function of shear rate at room temperature is shown in Figure 2(a) where the prepared starch adhesives exhibited the more pronounced non-Newtonian behavior. A shear-thinning behavior followed by shear-thickening was observed for all three compositions. The highest viscosity of the starch adhesive was noticed with 4.5 g mass concentration followed by 3.5 and 2.5 g. The critical shear rate at which the transition from shear-thinning to shear-thickening occurred was about 45.8 s^−1^ for adhesive having 2.5 g borate, 67.3 s^−1^ for adhesive having 3.5 g borate, and 78 s^−1^ for adhesive having 4.5 g borate. The borate was used as a solution thickener/stabilizer and to attain the required viscosity and solution structure. An increase in mass concentration of the borate strengthened the water holding and thickening ability of the adhesives. Nevertheless, the transition from shear-thinning to shear-thickening of adhesives occurred at low shear rates. It reveals that the adhesive rigidity increases as the shear rate surpasses the certain critical value which might be due to uniform mixing at high shear rates and/or swelling of the dispersion. Normally, the starch complexes exhibit shear-thickening behavior when the starch granules are rigid enough to resist the applied shear or the concentration of the gelling agents is high enough for the occurrence of particle crowding [13].

The temperature effects on the viscosity of the starch dispersions were also studied in these investigations as shown in Figure 2(b). The viscosity plotted against the shear rate for different constant temperatures of the starch adhesive (4.5 g borate) revealed that the viscosity was lowered with rise in temperature for all three mixing ratios. It was also noticed that shear-thickening response of the adhesives to the applied shear rate was undermined at high temperature values. At 80°C, the shear-thinning character was dominant as compared to shear-thickening as shown in Figure 2(c). After reaching the lowest viscosity values at critical shear rates, the viscosity was reached to a nearly constant state above 80°C. A similar trend was observed for all three compositions. Furthermore, the apparent viscosities of all mixing ratios of borate were found in close proximity at higher shear rates. With a rise in temperature and treatment time, the starch granules soften and become more deformable to the applied shear forces which results in a decrease in shear-thickening behavior. At higher temperatures, the shear-thinning to shear-thickening transition was occurring at lower shear rate.

#### 3.1.3. Surface Tension and Density

**Figure 3:**
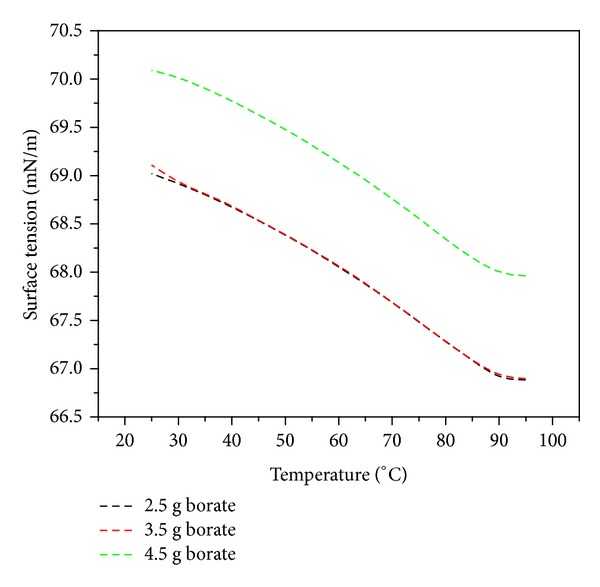
Starch adhesive surface tension as a function of heating temperature for different borate concentrations.

**Figure 4:**
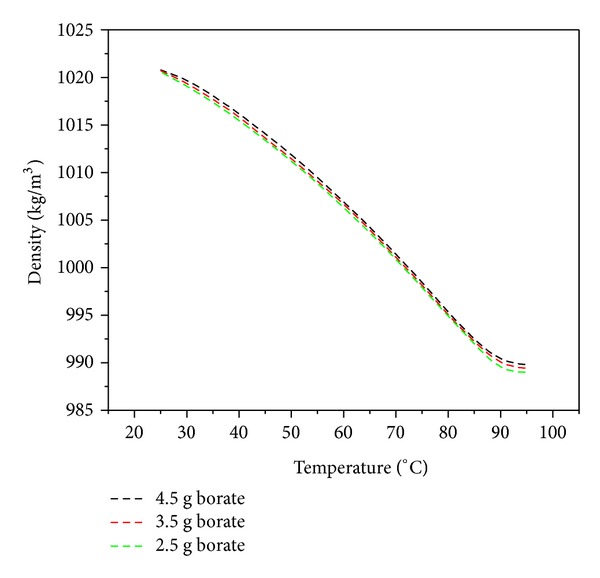
Starch adhesive density as a function of heating temperature for different borate concentrations.

In addition to the viscosity, the surface tension and density of the prepared adhesives were also measured as a function of temperature. A decreasing trend was observed in the surface tension plot with increasing temperature of the adhesive as shown in Figure 3. Above 90°C, the surface tension started achieving the constant values for all three mixing ratios. Furthermore, the surface tension of the adhesive with 4.5 g borate was noticeably higher than the other two compositions which were showing very close agreement for all temperature values. It has been reported that an increase in the borate mass ratio also enhances the molecular weight and water holding of starch adhesives [14]. It means that an increase in the borate mass concentration will also increase the density of the starch adhesives. But the results of these investigations showed a nominal effect of modifier mass concentration on the adhesive density as shown in Figure 4. Nevertheless, the influence of the temperature on the density was very prominent and a concomitant decrease in density with rise in temperature was noticed in these investigations.

### 3.2. Adhesive Spray Properties

**Figure 5:**
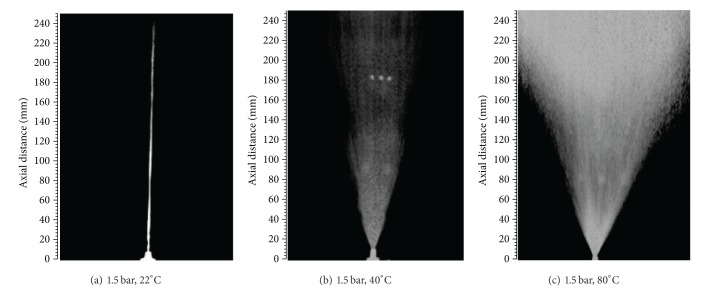
Images of the developing spray after 90 ms of jet injection time at 1.5 bar gauge pressure and different constant temperatures.

**Figure 6:**
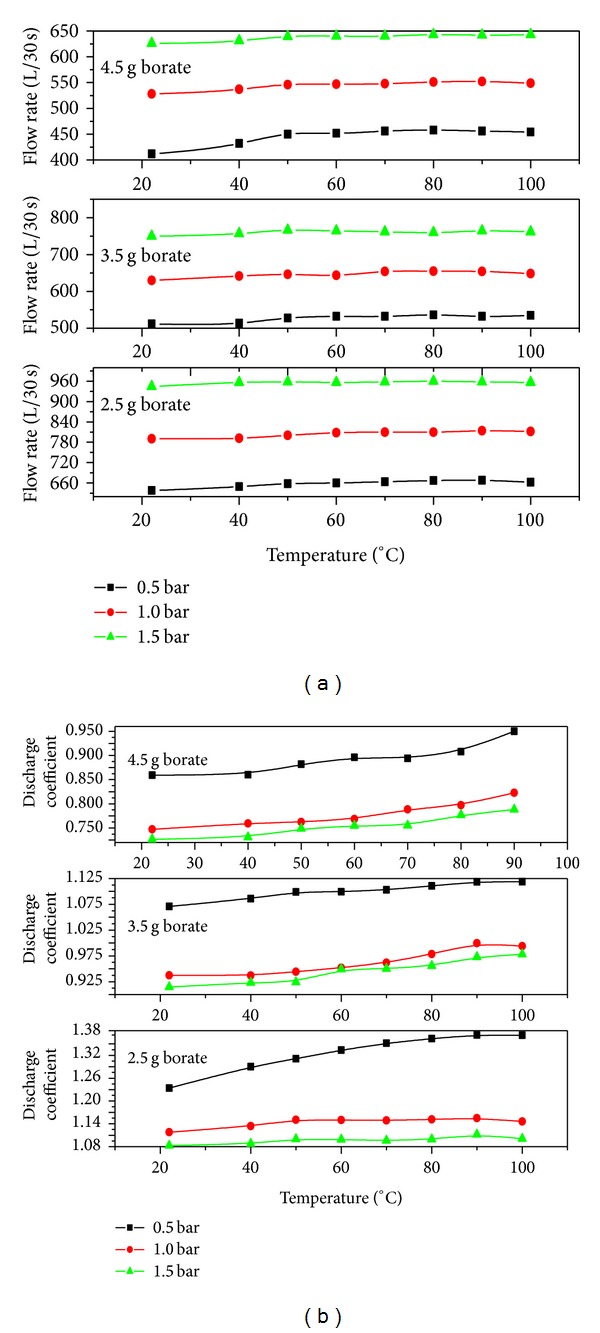
(a) Water flow rate and (b) nozzle discharge coefficient as a function of temperature for different pressures.

The visual and flow characterization of the sprays of starch adhesives energized by axisymmetric full cone atomizer and associated mechanism were conducted using a nonintrusive visualization technique. Some of the selected images grabbed at 1.5 bar load pressure and at different constant temperatures are presented in Figure 5. In this study, the spray patterns of all three compositions were investigated in the pressure range of 0.5–1.5 bar and temperature range of 20–100°C. The most promising results were obtained above 1.5 bar load pressure and 80°C heating temperature. The first section discusses the results of some of the adhesive flow parameters as a function of temperature, pressure, and time. The mass flow rates of the tested compositions are shown in Figure 6(a). The mass flow rate through the atomization facility was varied between the tested starch adhesives. Initial tests were performed using tap water to confirm the constant mass flow rate and to set a standard for judging any observed effects. Then non-Newtonian starch adhesives were run through the atomizer to investigate the pressure and temperature effects on the spray characteristics. Simply by increasing these input parameters, marked variations were observed in mass flow rate. It was noticed that the solution viscosity was decreased and the mass flow rate was initially increased with a rise in pressure and temperature and then reached a steady state after 50°C. The overall temperature effects on this flow parameter were significant at fixed low pressure values and 0.5 bar in particular. Overall, the starch adhesive with 2.5 g borate had the highest mass flow rate followed by 3.5 and 4.5 g.

The nozzle orifice only works well under the fully developed liquid flow profiles. With higher load pressures, the instabilities occur in the water stream. These instabilities are caused by the local vorticities in the liquid flow. Therefore, the equation of the density dependent mass flow rate within the nozzles can be expressed as [15]

(2) $$\begin{array}{c} \dot{m}=C_{D}A\cdot \sqrt{2\cdot \rho \left(p\right)}, \end{array}$$

where *ρ* is the water density, *Q* is the volumetric flow rate, *C*_*D*_ is the discharge coefficient, *A* is the cross-sectional area of the orifice, and *p* is the pressure difference. The derivation of the above equation involves the nozzle orifice opening area, and the use of such small cross-sectional areas at the vena contracta is not the realistic approach. In addition, the nonnegligible fractional forces, turbulence, and viscosity effects may also have the adverse effect on the mass flow rates. Therefore, the term discharge coefficient was introduced in these investigations. The discharge coefficient is the ratio of the mass flow rate at the discharge end of the nozzle to that of an ideal nozzle. The discharge coefficient as a function of injection pressure and temperature is shown in Figure 6(b), where a decreasing trend was observed in the discharge coefficient with an increase in injection pressure for all tested compositions. With increase in injection temperature, a steadily increasing trend similar to mass flow rate and mean flow velocity was observed in flow rate. It was predicted that the starch adhesive with 2.5 g borate had the highest discharge coefficient followed by 3.5 and 4.5 g mass ratios.

**Figure 7:**
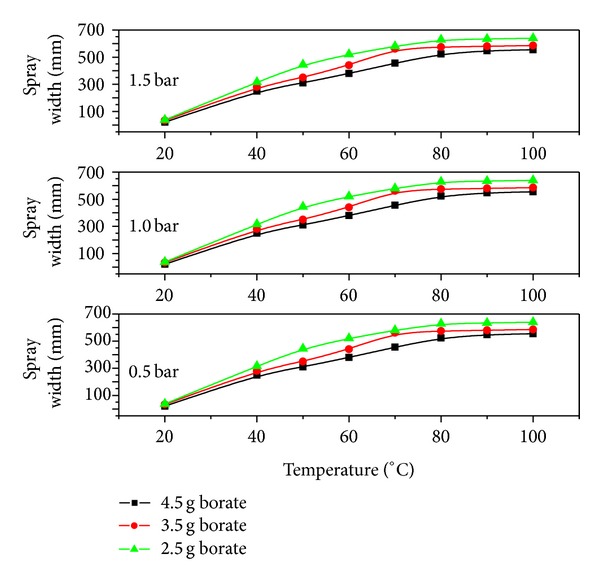
Spray cone width as a function of temperature for different pressures.

Normally, partial evaporation of the liquids in the system is stimulated by introduction of thermal energy below the liquid boiling point. The obtained vapor contents depend on the process parameters like the degree of heating, pressure, and nozzle geometry. When the liquid is discharged into the surrounding environment with a phase inversion, the jet disintegration takes place and the vapor phase inside the nozzle supports the disintegration process [16]. Therefore, in comparison with highly pressurized atomization, uniform spray patterns came out with a steadily increasing spray width (Figure 7) and constant spherical droplet sizes.

**Figure 8:**
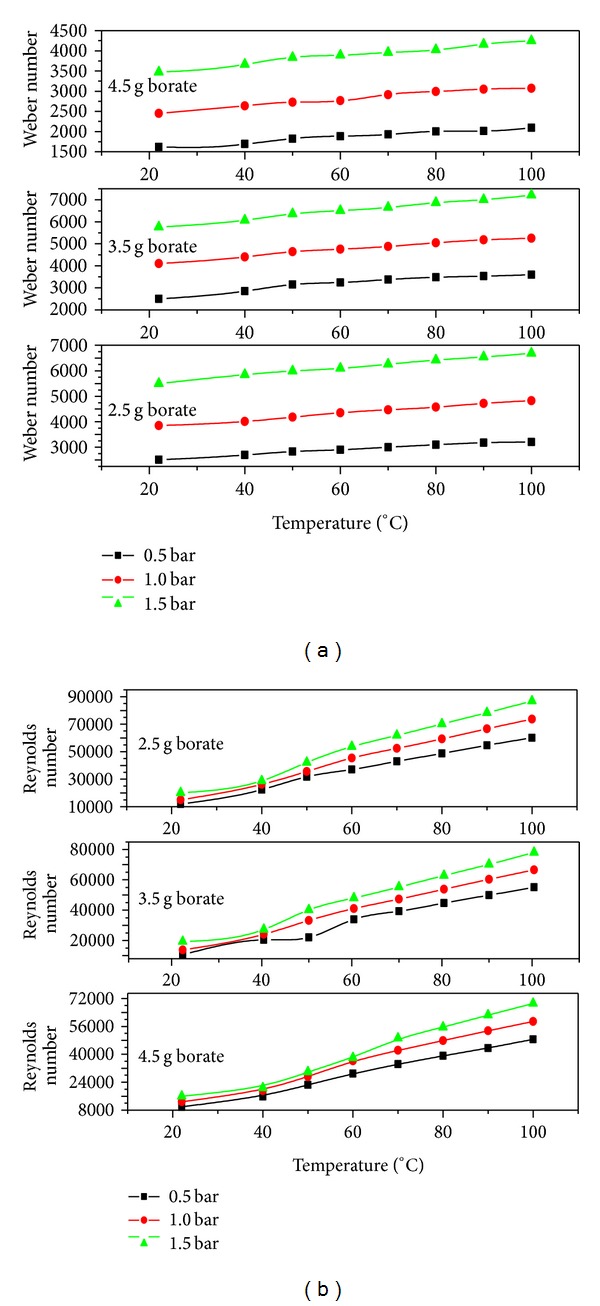
(a) Weber number and (b) Reynolds number as a function of temperature for different pressures.

Apart from the parameters discussed so far, the other flow regimes can also be achieved by use of heated liquids as spraying media [17]. The most critical spray parameters involve the liquid flow within the atomizer and the interaction between the liquid jet and the ambient air. The liquid flow within the actuator and atomizers can be described by dimensionless quantities called Weber and Reynolds numbers. Figures 8(a) and 8(b) show a monotonic increase both in Weber and Reynolds numbers which lead to an improved atomization and macroscopic spray cone length. The Reynolds number determines whether the liquid flow was dominated by inertial or viscous forces and hence either the flow was laminar or turbulent. The Reynolds number normally describes three mechanisms of liquid jet breakup [15]. Firstly, at low Reynolds numbers, large sized droplets come out according to the Raleigh mechanism of jet breakup. Secondly, at intermediate Reynolds numbers, the breakup is achieved by jet oscillations with respect to the jet axis until the jet disintegrates into ligaments and then small droplet [18]. The second regime produces a wide range of droplet sizes. Finally, at high Reynolds numbers, the complete atomization of the jet is achieved within a short distance from the orifice as in case of 2.5 g borate which had the highest Reynolds number among all the tested compositions.

The dependence of secondary atomization on relative velocities, liquid physical properties, and nozzle design is described by the Weber number. The Weber number is the ratio of the inertia of a fluid to its surface tension, therefore, the further droplet breakup and secondary atomization are expected to be strongly dependent on the Weber number [19]. In these investigations highest Weber number values were observed with 2.5 g borate which had the highest Weber number followed by 3.5 g and 4.5 g borate.

**Figure 9:**
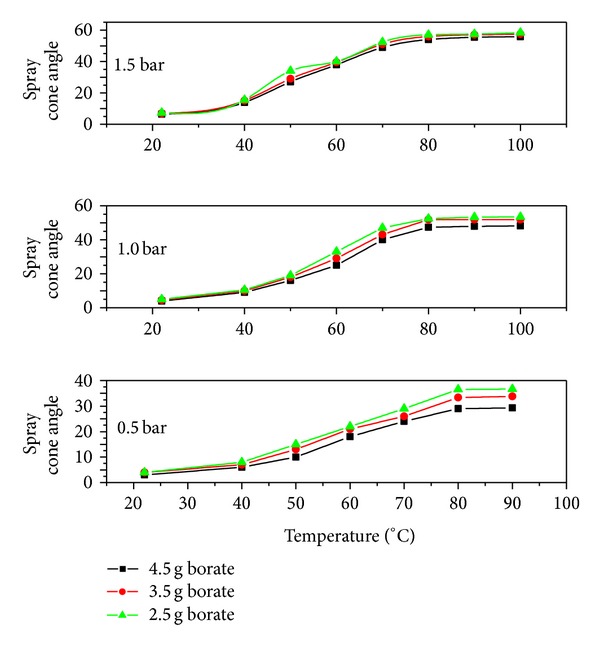
Spray cone angle as a function of temperature for different pressures.

The spray cone angle which defines the spray boundary was also measured against temperature by taking the mean value of 15 images as shown in Figure 9. No variation was observed in the cone angle for 0.5 bar load pressure while a slight incremental trend was seen at 1 and 1.5 bar pressures. Since, the spray cone angle investigations are usually carried out in a fully atomized mode, therefore, a decrease in the Weber number for a constant Reynolds number causes the spray cone angle to increase. This increase was considerably large at higher Weber numbers while for low Weber numbers, the curves showed steady nature. So, it can be concluded that at high Weber number values, the spray cone angle becomes less dependent on the Weber number [20]. During spray patterns visualization, it was observed that the main droplet streams divergence in the boundary ranging from 3 to 57.5° and if the droplet stream starts to shift its parameters out of this range then it will be the sign of the malfunction or nozzle damage. Therefore, it is advisable that for each flow condition, the spray visualization and the data analysis should be performed 5 times at least in order to assure the accuracy of the results obtained for the spray cone angle. In these studies, the maximum 6.7% error was noticed in spray cone angle while performing the error analysis.

**Figure 10:**
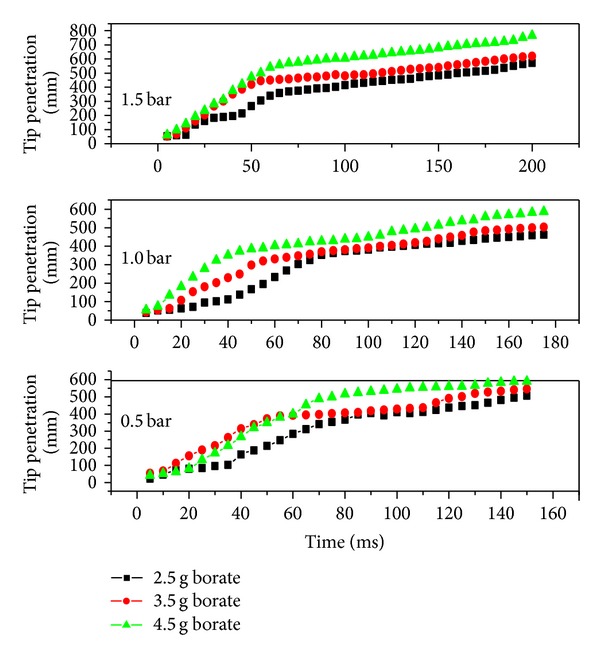
Spray tip penetration as a function of injection time for fixed temperature of 80°C.

The liquid tip penetration which is defined as the distance from the nozzle exit for a particular time interval was also measured as a function of temperature, time, and pressure.  Figure 10 depicts the tip penetrations with time at 80°C heating temperature. It was observed that the tip penetration increases with load pressure and time [21]. Up to 60 ms, there was a linear increase in tip penetration and thereafter a plateau. This steady increasing trend reveals that the evaporation of the medium varies with thermal energy injection and therefore consequent tip penetrations [22].

## 4. Conclusions

This paper reports the results of preparation and characterization of starch adhesives for future coating applications. The tapioca starch has successfully been modified by addition of different masses of borate as a gelling agent and urea as a cross-linker. Possible improvements in the physical properties of the synthesized adhesives have been presented by studying the effects of the temperature, shear rate, and mass of the borate on the density, viscosity, and surface tension of the adhesives. Then an attempt was made to relate the nozzle design, input parameters, and adhesive physical properties to the observed behavior of the spray patterns. The synthesized tapioca starch adhesives were atomized into full cone spray patterns by using an in-house built bottom to top spraying system. The adhesive formulations were tested at the temperature ranging from 20 to 100°C and the load pressure ranging from 0.5 to 1.5 bar. The spray imaging was carried out by using a high speed digital camera. The photographic studies confirmed that the adhesive temperature significantly influences the jet breakup and spray behavior in the ambient air. The injection of the thermal energy into the system appears to be the most important spray boundary condition which strongly affects the dimensionless parameters like Weber and Reynolds numbers. The most favorable results for these parameters were obtained at 1.5 bar load pressure and 80°C water temperature. The Weber and Reynolds numbers were also considered to be most relevant to the liquid jet breakup and evaporation because they create a delicate balance among the viscosity and retention for provision of very good atomization conditions. Finally, it was concluded that the adhesive formulation, the liquid delivery pump, spray nozzle, and the atomizing mechanism should always be well optimized for appropriate atomization outcomes.

## Figures and Tables

**Table 1:** The reaction conditions and mass concentration of materials.

Water (mL)	Starch (g)	Urea (g)	Borate (g)	Reaction temp. (°C)	Stirring rate (rpm)
1000	50	15	2.5	80	600 ± 5
1000	50	15	3.5	80	600 ± 5
1000	50	15	4.5	80	600 ± 5
